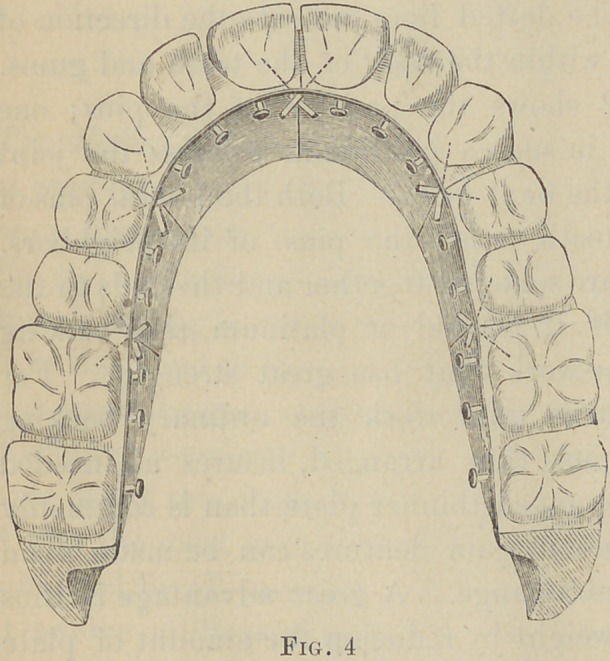# Improvement in Plate Teeth

**Published:** 1882-12

**Authors:** 


### EDITORIAL.

### Improvement in Plate Teeth.

Dr. C. N. Land, of Detroit, Michigan, has within the last year made a decided improvement in artificial teeth, which consists mainly in the insertion of three pins arranged transversely in the cervical part of the tooth, one in the center and one upon either lateral border of the tooth. They are made of smaller wire than the ordinary pins, and are longer than even those of the ordinary continuous gum teeth. These teeth are adapted for almost every kind of work, continuous gum, gold plate, celluloid and rubber work.

The accompanying illustrations will serve, with a few words, to convey a correct idea of the application of this improvement.

FIG. 1.

FIG. 1 presents the ordinary gum section of three teeth with the usual arrangement of the pins. The dotted lines indicate the direction of the pins within the body of the teeth and gums.

FIG. 2 shows the position of the pins; one long pin at each side, set in such a position as to cross the joint and also cross the pin of the next tooth. Both the lateral pins of each tooth cross the pins of its neighbors. These are soldered together and the ends to the plate, if it is gold or platinum, thus making a frame-work that has great strength. For continuous gum work the ordinary backing is not required, as the pins thus arranged, insures all needed strength, and that too with much thinner plate than is commonly used. Very strong continuous gum dentures can be made upon plate of No. 32 to 33 Stub's gauge. A great advantage is thus obtained in lessening the weight by reducing the amount of plate and body as well. The weight of the ordinary continuous gum work is in some instances at least a seeming objection. Another advantage is found in the greater ease and certainty with which the body and enamel is manipulated throughout.

FIG. 2.

The same arrangement for mounting on gold plate with rubber or celluloid is practicable. And for rubber or celluloid alone this arrangement of the pins can be employed equally as well if not better than those in common use. It will at once be seen the teeth thus arranged are mutually supporting, and afford great security against plates being split by pressure in ordinary use. Fig. 3 illustrates ten teeth united by the pins being crossed and soldered to each other.

Fig. 3.

Fig. 4 represents a set of teeth, some with the old and some with the new pins, arranged on the plate with the view of illustrating the great facility of giving increased strength by the use of the new pins. They cross, unite and give strength at all weak points, in a manner far superior to the pins in common use. This improvement is certainly worthy the careful consideration of the profession. These teeth will very soon be in the market. There will be no patent in connection with these teeth nor the process of mounting, to annoy the profession. Dr. Land was a little too discreet to place a patent between himself and the profession in this matter.

Fig. 4

---

It is an encouraging sign of the times that influential persons, in all parts of the country, are becoming more and more interested in the advancement of sanitary science.

## Figures and Tables

**Fig. 1. f1:**
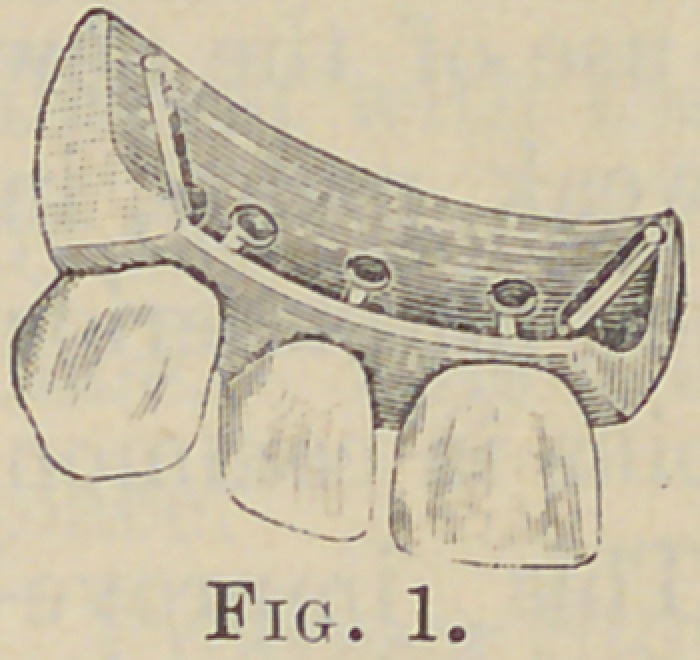


**Fig. 2. f2:**
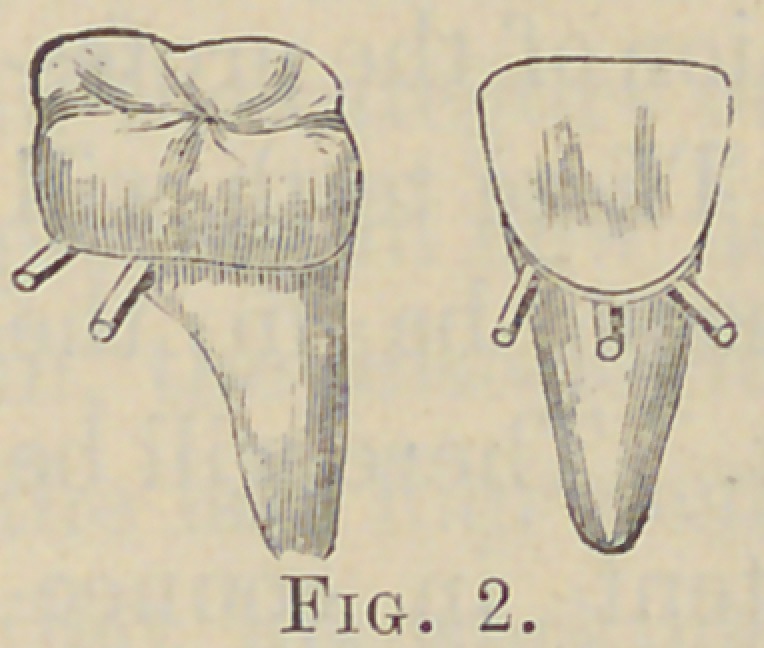


**Fig. 3. f3:**
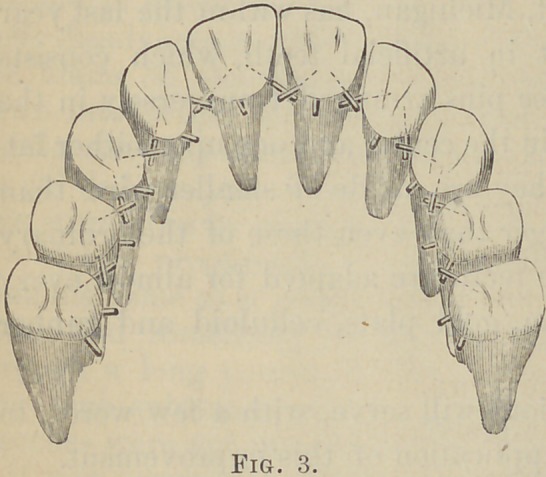


**Fig. 4. f4:**